# Glucose-to-lactate ratio and neurodevelopment in infants with hypoxic-ischemic encephalopathy: an observational study

**DOI:** 10.1007/s00431-022-04694-3

**Published:** 2022-12-09

**Authors:** Alfonso Galderisi, Mattia Tordin, Agnese Suppiej, Elisa Cainelli, Eugenio Baraldi, Daniele Trevisanuto

**Affiliations:** 1 Department of Woman’s and Child’s Health, University of Padova, Padova, Italy; 2 Division of Woman’s and Child’s Health, University Hospital of Padua, Padua, Italy; 3 Department of Medical Sciences-Pediatric Section, University of Ferrara, Ferrara, Italy; 4 Department of General Psychology, University of Padova, Padova, Italy

**Keywords:** Therapeutic hypothermia, Hypoxic-ischemic encephalopathy, Glucose-to-lactate ratio

## Abstract

We aimed to assess the glucose and lactate kinetics during therapeutic hypothermia (TH) in infants with hypoxic-ischemic encephalopathy and its relationship with longitudinal neurodevelopment. We measured glucose and lactate concentrations before TH and on days 2 and 3 in infants with mild, moderate, and severe hypoxic-ischemic encephalopathy (HIE). Neurodevelopment was assessed at 2 years. Participants were grouped according to the neurodevelopmental outcome into favorable (FO) or unfavorable (UFO). Eighty-eight infants were evaluated at follow-up, 34 for the FO and 54 for the UFO group. Severe hypo- (< 2.6 mmol/L) and hyperglycemia (> 10 mmol/L) occurred in 18% and 36% from the FO and UFO groups, respectively. Glucose-to-lactate ratio on day 1 was the strongest predictor of unfavorable metabolic outcome (OR 3.27 $\pm$ 1.81, *p* = 0.032) when adjusted for other clinical and metabolic variables, including Sarnat score.

*Conclusion*: Glucose-to-lactate ratio on day 1 may represent a new risk marker for infants with HIE undergoing TH.

**Table:** 

**What is Known:** *• Glucose and lactate are key metabolic fuels during neonatal hypoglycemia. This suggests that their concentrations may influence the neurodevelopmental outcome of neonates experiencing hypoxic-hischemic encephalopathy (HIE).*	
**What is New:** *• We describe the relative availbility of glucose and lactate before and during theraputic hypothermia in neonates with HIE.*

**Supplementary Information:**

The online version contains supplementary material available at 10.1007/s00431-022-04694-3.

## Introduction

Almost four out of 10 infants receiving therapeutic hypothermia (TH) for hypoxic-ischemic encephalopathy (HIE) experience one or more episodes of hypoglycemia, with the first episode occurring mainly during the first 24 h [1]. Hypoglycemia is thereby associated with more severe brain injuries on MRI and higher odds for adverse neurodevelopmental outcome [1] and reduces the neurodevelopmental benefits for those undergoing TH [2].

The combined hypoxic-ischemic and hypoglycemic damages during the first days of birth may explain the mechanism underlying these findings; however, alternative metabolic fuels can be used by the neonatal brain during hypoglycemia. In the presence of adequate oxygen delivery, lactate is produced by brain astrocytes and fuel neuronal brain metabolism [3, 4]. Lactate has been shown to increase during neonatal hypoglycemia in term babies on day 1, while ketone bodies (beta-hydroxybutyrate) seem to prevail on days 2, 3, and 4 [5] of birth, supporting their role as alternative brain fuels [6].

The availability of alternative brain fuels and their utilization may largely be affected by low blood cerebral flow and hypoxia occurring in term neonates with HIE; additionally, TH may itself favors hypoglycemia in the presence of relatively high lactate concentration, especially during day 1 of birth consequent to the hypoxic-ischemic damage [1].

The metabolic changes occurring during the first 6 h after HIE may influence the post-HIE recovery, and therefore, the risk index of neurodevelopmental impairment, representing a useful prognostic index, and identify those who would benefit from TH even among infants presenting with signs of mild encephalopathy.

Herein, we aimed to investigate the relative availability of glucose and lactate in a cohort of term neonates undergoing TH for HIE and the effect of early metabolic pattern on long-term neurodevelopmental outcome.

## Methods

We designed a retrospective study aimed to evaluate glucose and lactate trajectory during TH and its relationship with longitudinal neurodevelopmental outcome. We analyzed data obtained from clinical records of term neonates admitted to the neonatal intensive care unit of the University Hospital of Padova for HIE receiving TH between 2009 and 2016. Inclusion criteria for TH [8] were gestational age ≥ 35 weeks and any of the following: arterial umbilical cord or first blood gas analysis (within 1 postnatal hour) pH ≤ 7.0, and base excess < 12, or 10-min Apgar score < 5, or need for respiratory support at 10 min of life; and moderate to severe encephalopathy within 6 h of birth defined according to Sarnat and Sarnat score [9]. TH was started within 6 h after birth and consisted of whole-body hypothermia (33.0–34.0 °C) for 72 h followed by a rewarming rate of approximately 0.5 °C per hour [10]. Participants with a Sarnat score of 1—mild encephalopathy—have been included if they underwent whole-body hypothermia during the study period. Arterial glucose and lactate were measured at the beginning of the cooling phase of TH (day 1) and on the morning arterial sample collected on days 2 and 3 of treatment. Samples were processed cot side using the Siemens AutomaticQC (Siemens Healthcare GmbH, Germany).

Mild and severe hypoglycemia were defined as glucose concentrations < 2.6 mmol/L (47 mg/dL) and < 5 mmol/L (72 mg/dL), respectively; mild and severe hyperglycemia were defined for value higher than 8 mmol/L (144 mg/dL) and 10 mmol/L (180 mg/dL), as previously described [11, 12].

Lactate concentrations were grouped by umbilical arterial reference centiles for healthy term infants, with 1.0, 1.7, 7.2, and 9.0 mmol/L representing the 3rd, 10th, 90th, and 97th centile [13, 14].

Participants were evaluated after 24 months for neurodevelopmental assessment with the Griffith scale. The scale evaluates five specific developmental domains: locomotor, personal-social, hearing and language, eye-hand coordination, and performance [15]. Unfavorable outcome (UFO) was defined in the presence of a Griffith score < 70 in one of the domains of the scale or if the infant was diagnosed with cerebral palsy or in the event of death. Favorable outcome (FO) was defined as the absence of all these events.

The study was approved by the Ethics Committee of the Azienda Ospedaliera di Padova, Padova, Italy (number 67575 RF-2009–1511075), and written informed consent was obtained from the parents. This cohort was partially described elsewhere with respect to the nephrological complications associated with HIE [10, 16].

### Statistical analysis

The analysis was stratified according to the binary neurodevelopmental outcome at 2-year defined as “favorable” (FO) or “unfavorable” (UFO) as described above. A subgroup analysis for the glucose-to-lactate ratio was conducted based on the Sarnat score at admission.

Adjusted analyses of the effect of glucose, lactate, and glucose-to-lactate ratio on the binary outcome (UFO vs. FO) were performed using logistic regression modelling. Each participant had 3 measures for glucose and lactate corresponding to day 1 (beginning of TH), day 2, and day 3 samples. The analysis included only data from the samples of those who returned for the follow-up assessment. Model covariates were selected according to previous evidence [17] and included Sarnat score; birth weight; gestational age; Apgar at 1, 5, and 10 min; necessity for mechanical ventilation during the first 3 days of birth; sex; and the glucose and lactate concentrations on days 1, 2, and 3. Before including the covariates into the final model, we examined them for multicollinearity. Statistical significance for the parameter estimates glucose-to-lactate ratio was established with alpha of 0.025. Results were summarized as odds ratios (ORs) and standard error (SE).

ROC area was computed for the glucose-to-lactate rate against the binary neurodevelopmental outcome (FO or UFO).

Statistical comparisons of the continuous variables’ distributions were conducted using the non-parametric Mann–Whitney test, with a critical level of alpha at 0.05. The results were reported as median (25th percentile, 75th percentile). Categorical variables were compared using the Chi-square test. Variability was expressed as standard deviation (SD) as necessary.

Analysis has been conducted only in participants who returned for the follow-up assessment at 2 years.

STATA.13 (StataCorp 2013; Stata Statistical Software: Release 13; College Station, TX) and GraphPad Prism 8.0 (GraphPad Software, San Diego, CA) have been used for the study analysis.

## Results

Eighty-eight neonates out of 117 (75%) returned for the follow-up assessment and were included in the final analysis (Supplemental Fig. 1). Thirty-four (39%) did not exhibit any neurodevelopmental impairment (FO group), while 54 (61%) experienced an adverse neurological outcome (UFO group), including a Griffith score < 70 in one of the domains (35/88) or the global score (15/88). Six participants (7%) were diagnosed with cerebral palsy, and 10 (11%) died before the 2-year evaluation. The two groups did not differ for baseline characteristics, including Apgar at 1, 5, and 10 min and Sarnat score (Table 1).

**Fig. 1:**
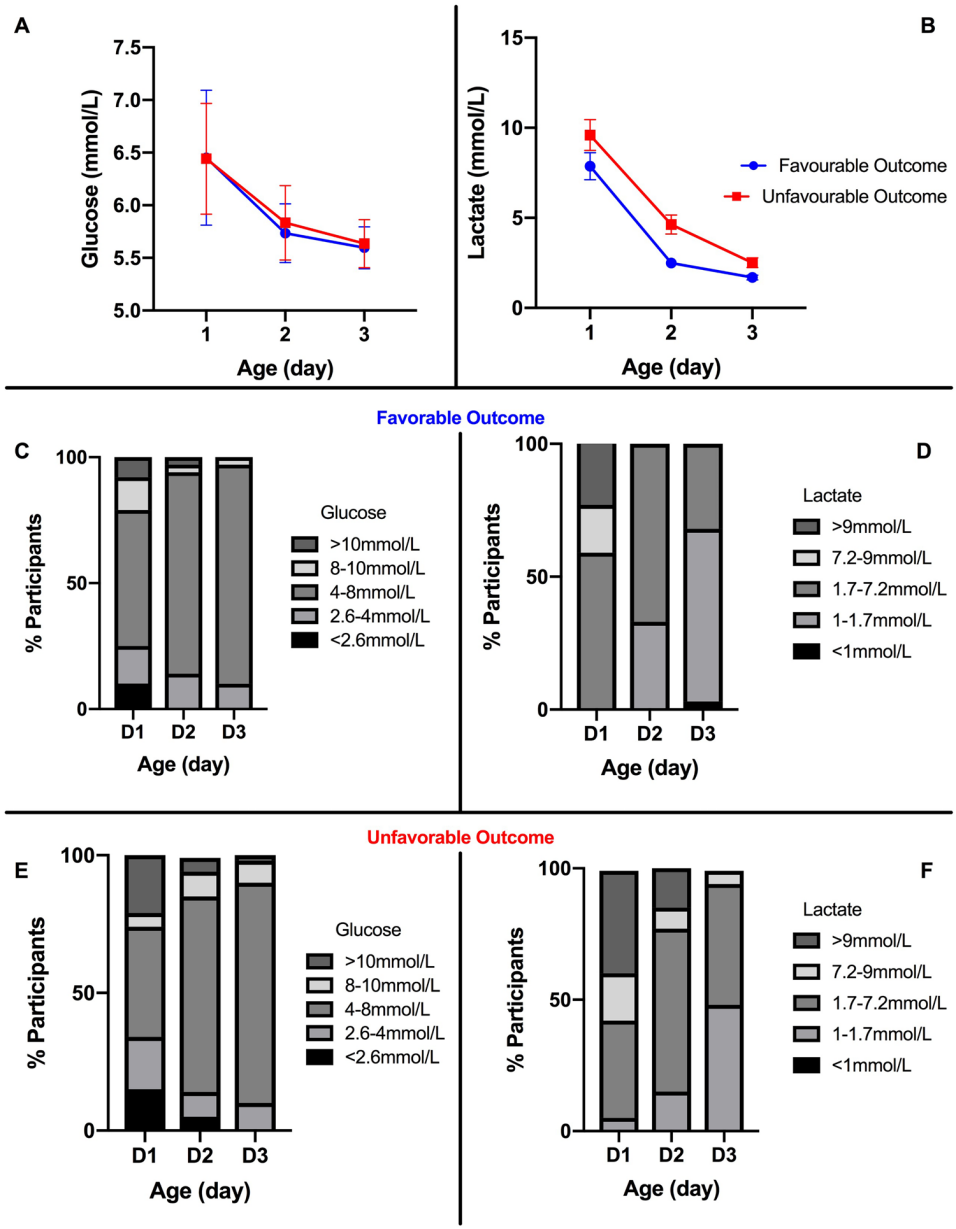
Glucose (**A**) and lactate (**B**) concentrations on days 1, 2 and 3 in those with unfavorable and favorable outcome. Percentage of participants in the prespecified glucose (**C**) and lactate (**D**) ranges in those with favorable outcome. Percentage of participants in the prespecified glucose (**C**) and lactate (**D**) ranges in those with unfavorable outcome

**Table 1:** Participants characteristics

	Favorable outcome (*n* = 34)	Unfavorable outcome (*n* = 54)	*p*
Gestational age (weeks)	39 (38, 40)	40 (38, 40)	0.455
Birth weight (g)	3280 (3000, 3640)	3300 (2975, 3740)	0.327
Sex, *n* (%)			
Apgar 1’	3 (2, 5)	2 (1, 5)	
5’	6 (4, 7)	5 (3, 7)	0.332
10’	7 (5, 8)	6 (5, 8)	
Sarnat and Sarnat			
1	13	14	
2	21	30	0.209
3	1	9	
Mechanical ventilation	26 (76)	54 (100)	0.149
Glucose D1 (mmol/L)	6.3 (3.9, 8.8)	4.9 (3.4, 8.3)	0.325
Lactate D1 (mmol/L)	6.9 (4.9, 9.7)	8.6 (4.4, 14.4)	0.184

Data are expressed as median (IQR) or number (percentage)

The median glucose and lactate concentrations on day 1 were similar between FO and UFO groups (*p* = 0.649 and *p* = 0.369) (Fig. 1A, B): 18% (6/34) of neonates in the FO group and 36% (19/54) of those with UFO exhibited a glucose concentration on day 1 out of the range 2.6–10 mmol/L, with similar prevalence of severe hypo- and hyperglycemia (12% (6/54) and 11% (4/34) participants experienced severe hypo in the UFO and FO group, respectively, while 26% (14/54) and 21% (7/34) had severe hyperglycemia) (*p* = 0.107). The prevalence of both hypo- and hyperglycemia dropped on days 2 and 3 in both groups (Fig. 1C, E).

Similarly to glucose fluctuations, lactate concentrations progressively decreased on days 2 and 3. While the percentage of participants with lactate concentrations higher than 1.7 mmol/L was similar in the two groups on day 1 (94% [51/54] and 97% [33/34] for the UFO and FO groups respectively, *p* = 0.163), the drop of lactate concentrations was faster in those with FO than in the UFO group with 41% [14/34] and 24% [13/54] participants from the FO and FO groups having a lactate concentration < 1.8 mmol/L on day 2 (*p* = 0.016) and 76% [26/34] and 54% [29/54] on day 3 (*p* = 0.037) (Fig. 1D, F).

### Glucose and lactate dynamics and neurodevelopmental outcome

**Fig. 2:**
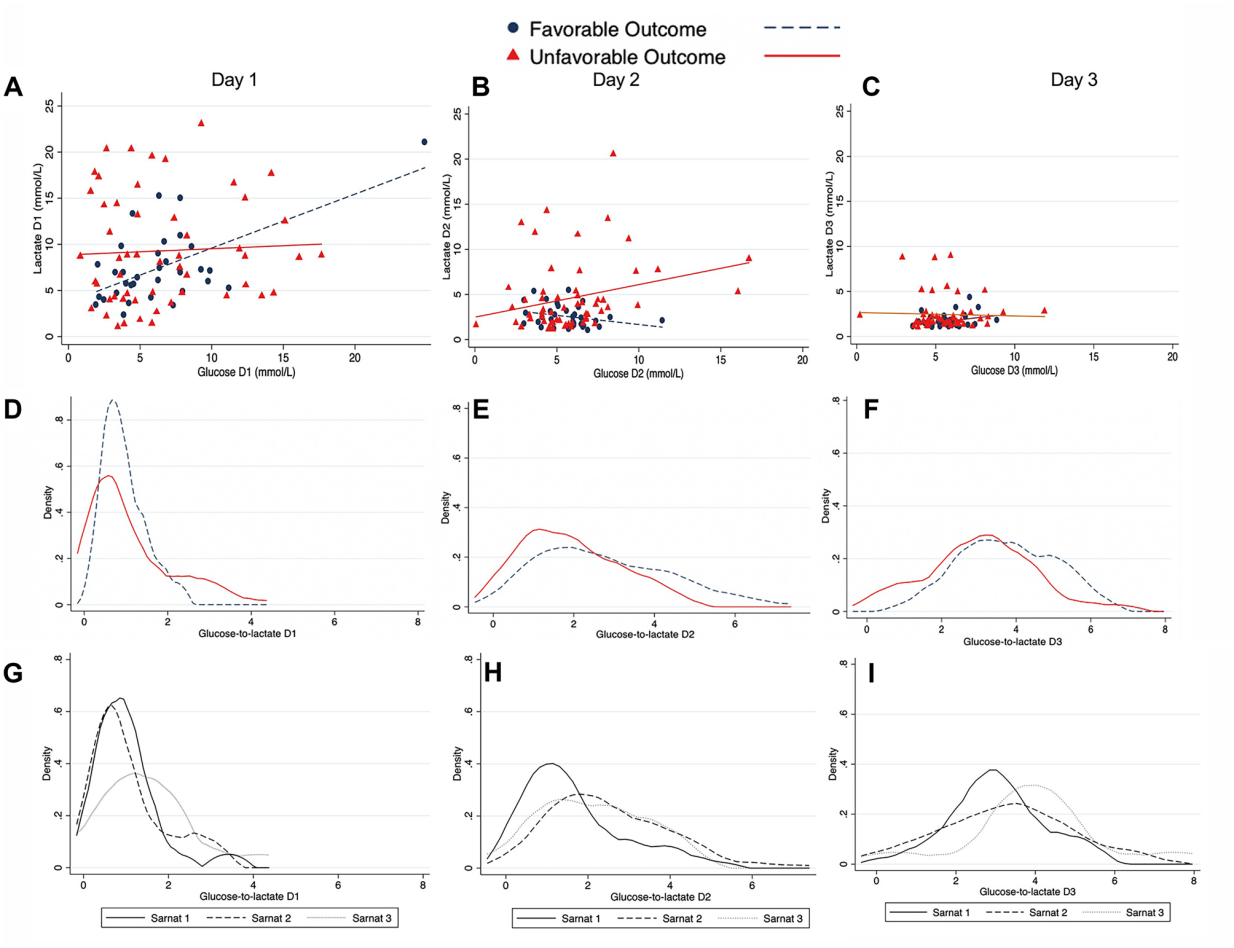
Glucose and lactate relationship on days 1 (**A**), 2 (**B**), and 3 (**C**) by neurodevelopmental outcome at 2 years. Distribution (density) of glucose-to-lactate ratio by neurodevelopmental outcome on day 1 (**D**), day 2 (**E**), and day 3 (**F**). Distribution (density) of glucose-to-lactate ratio by Sarnat score on day 1 (**G**), day 2 (**H**), and day 3 (**I**)

The relationship between glucose and lactate was then explored according to the longitudinal neurodevelopmental outcome. We observed a linear relationship between the two metabolites ($r$= 0.6, *p* < 0.001) in those with a favorable outcome on day 1, while lactate exhibited limited changes upfront glucose concentrations in the group with unfavorable outcome ($r$= 0.14, *p* = 0.731) (Fig. 2A).

On day 2, we did not observe a significant association between glucose and lactate neither in those with favorable outcome ($r$= 0.28, *p* = 0.108) nor in the UFO group ($r$= 0.26, *p* = 0.786) (Fig. 2B). The absence of an association was confirmed on day 3 ($r$= 0.32, *p* = 0.057 and $r$= 0.11, *p* = 0.801 for the FO and UFO group, respectively) (Fig. 2C).

The distribution of the glucose-to-lactate ratio by neurodevelopmental is displayed in Fig. 2 for each study day and confirms a narrow distribution of the ratio values on day 1 for the FO group vs. the UFO (SD 0.48 vs. 0.99) that becomes wider over days 2 (SD 1.7 vs. 2.8) and 3 (SD 1.2vs 1.8) (Fig. 2D–F).

The daily distribution of the glucose-to-lactate ratio was similar for the Sarnat 1 and 2 regardless of the neurodevelopmental outcome, while those with Sarnat 3 exhibited a greater glucose-to-lactate ratio on day 1 (Fig. 2G) than the other two groups. Glucose-to-lactate profiles of those with Sarnat 1 and 2 were similar throughout the study period (Fig. 2H, I).

### Early metabolic risk determinants of neurodevelopmental outcome

**Fig. 3:**
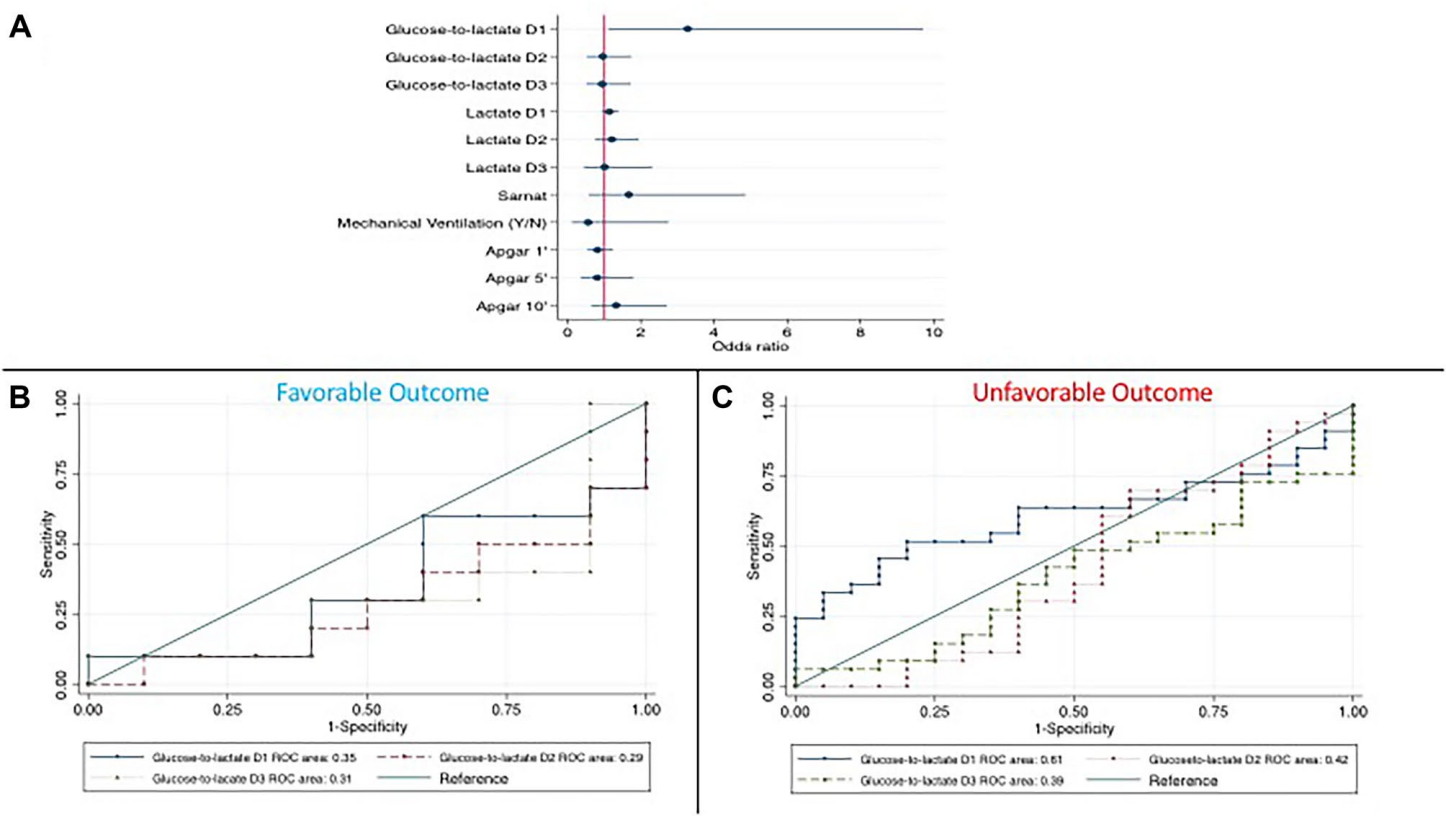
**A** Odds ratio for unfavorable outcome at 2-year. **B** Receiver-operating curve (ROC) for glucose-to-lactate ratio for the binary outcome neurodevelopment at 2 years (favorable or unfavorable) using the glucose-to-lactate ratio on days 1, 2, and 3 in those with a favorable (**B**) or unfavorable (**C**) outcome

Thereby, we explored the effect of glucose-to-lactate ratio on days 1, 2, and 3 and its components on the neurodevelopmental outcome modelled with respect to other clinically relevant variables. The glucose-to-lactate ratio on day 1 resulted to be the strongest predictor of unfavorable neurodevelopmental outcome at 2 years (*p* = 0.018) with an odds for the unfavorable outcome 3 times higher for each unitary increase of the glucose-to-lactate ratio [OR 3.28 $\pm$ 1.8, *p* = 0.032) (Fig. 3A) when the other variables were held constant. Such an association was not observed on days 2 and 3, nor for the glucose and lactate concentrations. The ROC area for glucose-to-lactate ratio on day 1 was 0.61 in the group with the unfavorable outcome and 0.35 in those with the favorable outcome when adjusted for the other modelled variables (Fig. 3B, C).

When the regression analysis was stratified by Sarnat score, a glucose-to-lactate ratio on day 1 higher than 1.28 resulted to predict 100% of the unfavorable outcome cases in the group with a Sarnat score of 3. Sarnat score demonstrated a weaker association with the neurodevelopmental outcome on a multivariate logistic regression analysis (OR 1.67 $\pm$ 0.9, *p* = 0.348).

## Discussion

We displayed glucose and lactate kinetics during the first 3 days of life of infants with HIE undergoing TH and the association between glucose-to-lactate ratio on day 1 with 2-year neurodevelopmental outcome.

First, our cohort confirmed that both severe hypo- and hyperglycemia are frequent in infants with HIE, as ~ 35% of infants with unfavorable outcome and 18% with favorable outcome exhibited glucose values out of the range 2.6–10 mmol/L. During TH, the prevalence of hypo- and hyperglycemia progressively diminished with no infant exhibiting glucose value out of the 2.6–10 mmol/L range on the last day of TH. The Sugar Babies study described that more than 50% of healthy term infants, at risk for hypoglycemia, experiences hypoglycemic events (< 2.6 mmol/L) in the absence of clinical signs [18, 19], while ~ 25% of infants from the UK TOBY cooling registry [20] had at least one hypoglycemic episodes during TH. Our cohort demonstrates that both hypo- and hyperglycemia can be observed on day 1 and their frequency is higher in those with unfavorable outcome. We adopted an operational definition of hypoglycemia (glucose concentration less than 2.6 mmol/L [11, 21], although we are aware that a lower threshold (1.9 mmol/L = 35 mg/dL) may be advised by the American Academy of Pediatrics before 24 h of birth [22, 23]. To this end, our data have been presented with respect to the percentage of patients in prespecified ranges as well as continuous measures; this latter is expected to overcome the conflictual definition of glucose thresholds. Even though we did not obtain serial samples nor adopted continuous glucose measures, the sampling frequency was regular with respect to time of the day (7–9 am in the morning) and sampling conditions; thus, the results could be easily compared. The sample on day 1 was collected right before the beginning of TH (< 6 h of birth).

Second, we explored the relative change of lactate during the first 3 days of life. Lactate is an alternative brain metabolic fuel in preterm and term infants [5, 24] and contributes to ~ 25% of the energetic production of term infants during the first day of life [24]. In healthy term infants, a linear relationship between glucose and lactate has been demonstrated during the first 12 h of life [24]. Herein, we demonstrated that such a relationship is preserved only in those with a favorable long-term outcome, while it is disrupted in infants with unfavorable outcome at 2 years. For the first time, we describe that the existence of such a metabolic kinetics between glucose and lactate is associated with a favorable outcome. Indeed, the unitary increase of glucose-to-lactate ratio on day 1 resulted into a 3 times higher odd for 2-year unfavorable outcome, out of several known risk determinants (including glucose and lactate alone, mechanical ventilation, and the Apgar score). A higher glucose-to-lactate ratio can result from isolated hyperglycemia unparalleled by a simultaneous increase of lactate concentration. In a post-hoc analysis from the CoolCap study, only children presenting glucose concentrations greater than 8.3 mmol/L exhibited a significant benefit for hypothermia with respect to the neurodevelopmental outcome [25]; however, lactate data and the glucose-to-lactate ratio were not available. A differential glucose-lactate kinetics might provide a more comprehensive figure of the metabolic pathways involved in the brain damage after HIE and of the effect of TH.

A higher glucose-to-lactate ratio does not necessarily derive from hyperglycemia, as the percentage of infants experiencing glucose values greater than 8 and 10 mmol/L was similar between the UFO and FO groups in our cohort. Lactate changes might be due to the presence of alternative metabolic pathway diverging lactate toward the pyruvate-ketone route that may prevail during the first hours after hypoxic injury or to the inability of peripheral tissues—brain and muscles—to generate lactate in the presence of mild or severe hyperglycemia [26, 27]. We can hypothesize that lactate may serve as an alternative brain fuel during hyperglycemia. Indeed, as post-HIE hyperglycemia is mainly hypo insulinemic, the brain glucose uptake is partially limited by the inability to mobilize insulin-dependent brain glucose transporter (GLUT1). Thus, lactate may easily serve as an alternative fuel for the hypoxemic neuronal tissue [4, 28], and a higher glucose-to-lactate ratio would be a marker of hyperglycemia in the absence of a compensatory lactate increase on day 1.

Third, our observations suggest that the glucose-to-lactate ratio on day 1 could stratify the risk for unfavorable outcome, with a stronger association than other known risk determinants, including the Sarnat score and the Apgar score as well as the need for mechanical ventilation or lactate concentrations [9, 29]. Interestingly, our cohort included a limited number of participants with mild encephalopathy (Sarnat score 1) that are not regularly offered TH, despite some evidence for a benefit deriving from TH in this group [7]. When the glucose-to-lactate ratio was stratified by Sarnat group, we observed that the kinetics of the two metabolites was similar in those with Sarnat 1 and 2, while the ratio greatly increased in those with Sarnat 3. This observation is suggestive—though not conclusive—for a metabolic proximity of infants with mild and moderate HIE and a greater difference for those with severe HIE with respect to the availability of metabolic substrates.

During TH, glucose and lactate concentrations tend to normalize. We did not observe any significant difference between the two metabolites and their relationship during days 2 and 3.

The major limitation of our study is the absence of a continuous measure of glucose and lactate that would have provided the time of metabolic brain exposure. Additionally, other metabolic fuels such as ketones may play a role after HIE and contribute to the observed inter-individual variability. The limited numerosity of the study prevents the generalizability of our results since we cannot identify additional determinants of the observed outcome including perinatal characteristics as well as social determinants that may in turn play a major role in a longitudinal study. A larger longitudinal multicenter study could provide the necessary evidence to introduce glucose-to-lacate ratio into the clinical practice of NICU during HIE treatment.

Our observations suggest a role for lactate on day 1 of life as a main determinant of the brain metabolism of infants with HIE; thus, the glucose-to-lactate ratio—instead of the individual concentrations—may represent a new marker for neurodevelopmental outcome in infants with HIE. Blood concentrations of lactate closely reflect brain concentration. After intravenous infusion, the increase of lactate has been shown to improve brain function in adults during hypoglycemia [29]. We can hypothesize that higher lactate levels may be neuroprotective after HIE. Such a goal might be achieved by interventions targeting early parenteral and enteral nutrition [30] that might be able to regulate lactate concentrations to reach neuroprotection.

## Supplementary Information

Below is the link to the electronic supplementary material.

Supplementary file1 (PDF 41 KB)

## Data Availability

Data are available upon reasonable request to the corresponding author.
