# Supplementary figures and images for: Glucose-to-lactate ratio and neurodevelopment in infants with hypoxic-ischemic encephalopathy: an observational study
Source: Eur J Pediatr. 2022 Dec 9;182(2):837–44. doi: 10.1007/s00431-022-04694-3 (PMC9899169; doi:10.1007/s00431-022-04694-3)

**Supplemental Figure 1. Consort flow diagram.**

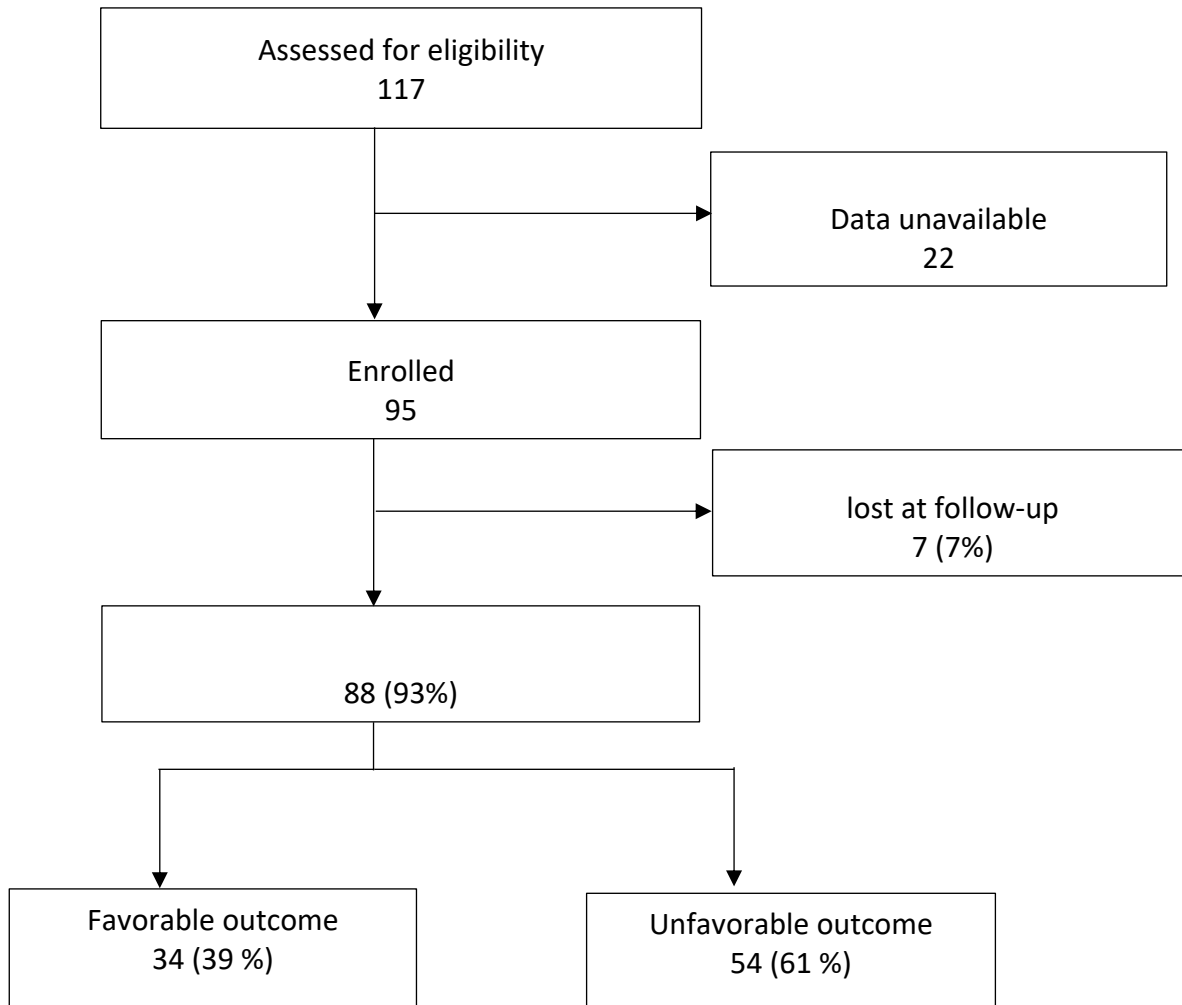

Supplement: Supplementary file 1 — Supplementary file1 (PDF 41 KB) [file 431_2022_4694_MOESM1_ESM.pdf]
